# SPARKI: a tool for the statistical analysis of pathogen identification results

**DOI:** 10.1093/bioinformatics/btaf596

**Published:** 2025-10-31

**Authors:** Jacqueline M Boccacino, Martin del Castillo Velasco-Herrera, Mathew A Beale, Jamie Billington, Ian Vermes, Sofia Obolenski, Kim Wong, Laura Torrens, Sarah Moody, Sandra Perdomo, Saamin Cheema, Bailey Francis, Victoria Offord, Adam P Butler, David J Adams

**Affiliations:** Cancer, Ageing and Somatic Mutation Programme, Wellcome Sanger Institute, Wellcome Genome Campus, Hinxton, Cambridgeshire, CB10 1SA, United Kingdom; Cancer, Ageing and Somatic Mutation Programme, Wellcome Sanger Institute, Wellcome Genome Campus, Hinxton, Cambridgeshire, CB10 1SA, United Kingdom; Parasites and Microbes Programme, Wellcome Sanger Institute, Wellcome Genome Campus, Hinxton, Cambridgeshire, CB10 1SA, United Kingdom; Cancer, Ageing and Somatic Mutation Programme, Wellcome Sanger Institute, Wellcome Genome Campus, Hinxton, Cambridgeshire, CB10 1SA, United Kingdom; Cancer, Ageing and Somatic Mutation Programme, Wellcome Sanger Institute, Wellcome Genome Campus, Hinxton, Cambridgeshire, CB10 1SA, United Kingdom; Cancer, Ageing and Somatic Mutation Programme, Wellcome Sanger Institute, Wellcome Genome Campus, Hinxton, Cambridgeshire, CB10 1SA, United Kingdom; Department of Dermatology, Leiden University Medical Centre, Leiden, 2333 ZA, The Netherlands; Cancer, Ageing and Somatic Mutation Programme, Wellcome Sanger Institute, Wellcome Genome Campus, Hinxton, Cambridgeshire, CB10 1SA, United Kingdom; Genomic Epidemiology Branch, International Agency for Research on Cancer (IARC/WHO), Lyon, 69366, France; Cancer, Ageing and Somatic Mutation Programme, Wellcome Sanger Institute, Wellcome Genome Campus, Hinxton, Cambridgeshire, CB10 1SA, United Kingdom; Genomic Epidemiology Branch, International Agency for Research on Cancer (IARC/WHO), Lyon, 69366, France; Cancer, Ageing and Somatic Mutation Programme, Wellcome Sanger Institute, Wellcome Genome Campus, Hinxton, Cambridgeshire, CB10 1SA, United Kingdom; Cancer, Ageing and Somatic Mutation Programme, Wellcome Sanger Institute, Wellcome Genome Campus, Hinxton, Cambridgeshire, CB10 1SA, United Kingdom; Cancer, Ageing and Somatic Mutation Programme, Wellcome Sanger Institute, Wellcome Genome Campus, Hinxton, Cambridgeshire, CB10 1SA, United Kingdom; Cancer, Ageing and Somatic Mutation Programme, Wellcome Sanger Institute, Wellcome Genome Campus, Hinxton, Cambridgeshire, CB10 1SA, United Kingdom; Cancer, Ageing and Somatic Mutation Programme, Wellcome Sanger Institute, Wellcome Genome Campus, Hinxton, Cambridgeshire, CB10 1SA, United Kingdom

## Abstract

**Motivation:**

Many pathogen identification and microbiome analysis tools have been developed in recent years, with Kraken 2 being one of the most popular. While tools downstream of Kraken 2 can assist in the interpretation of its outputs, a statistical framework to assess the likelihood that a taxon/organism is present in a single sample alongside an automated end-to-end analysis pipeline has not yet been fully implemented.

**Results:**

Here, we introduce SPARKI, an R package that performs statistical analysis of Kraken 2 outputs and aids in the identification of pathogens present in next-generation sequencing samples. SPARKI adds to the field by bringing a probabilistic view to Kraken 2 data, serving as a discovery tool and complementing other methods such as KrakenTools, Bracken, and Pavian.

**Availability and implementation:**

SPARKI code is available on GitHub at https://github.com/team113sanger/sparki. SPARKI is also part of an end-to-end pathogen identification pipeline, sparki-nf, which is available at https://github.com/team113sanger/sparki-nf. An additional pipeline for further exploration and validation of SPARKI results is also available at https://github.com/team113sanger/map-to-genome.

## 1 Introduction

Pathogen identification and microbiome analysis have become increasingly prominent in many research areas. Several tools have been developed to help tackle this challenge, including Kraken 2 (Wood *et al.* 2019), a *k*-mer-based tool that classifies sequencing reads taxonomically. Briefly, Kraken 2 splits the sequencing reads from a FASTQ file into *k*-mers, from which substrings denominated minimizers are obtained. By calculating a compact hash code for each minimizer, Kraken 2 is then able to efficiently access a database of taxa and assign each *k*-mer the appropriate lower common ancestor taxon. When run with the ‘--report’ option, Kraken 2 generates a sample report containing all taxa, at different taxonomic ranks, which are identified in the sample. If run with the flag ‘--report-minimizer-data’, Kraken 2 also outputs the number of unique minimizers associated with each taxon that were found in a sample.

While Kraken 2 is a popular tool for pathogen identification and microbiome analysis, its outputs can be ambiguous. Minimizers can be misattributed to taxa—with attributions being impacted by the number and the composition of the query sequences provided—and a statistical framework has not yet been fully implemented in the software to quantify the likelihood that a taxon/organism is truly present in a sample. Users can adjust the confidence score of their Kraken 2 analysis by modifying the ‘--confidence’ parameter to reduce the risk of false positive taxa discoveries, but this approach will not report the statistical significance—and thus the surety—with which a taxon is identified.

Several additional tools—such as KrakenTools (Lu *et al.* 2022), Bracken (Lu *et al.* 2017), and Pavian (Breitwieser and Salzberg 2019)—can be used for downstream processing of Kraken 2 results. KrakenTools, e.g. provides a set of scripts to perform operations on results generated by Kraken 2, such as read extraction and report collation; Bracken leverages the Bayes’ theorem to re-estimate taxa abundance for lower taxonomic ranks, such as genus and species, and obtain outputs with a higher accuracy; and Pavian allows users to identify potentially interesting results based on the number of reads assigned to each taxon, which can be visualized in terms of percentages or *z*-scores.

**Figure 1.**
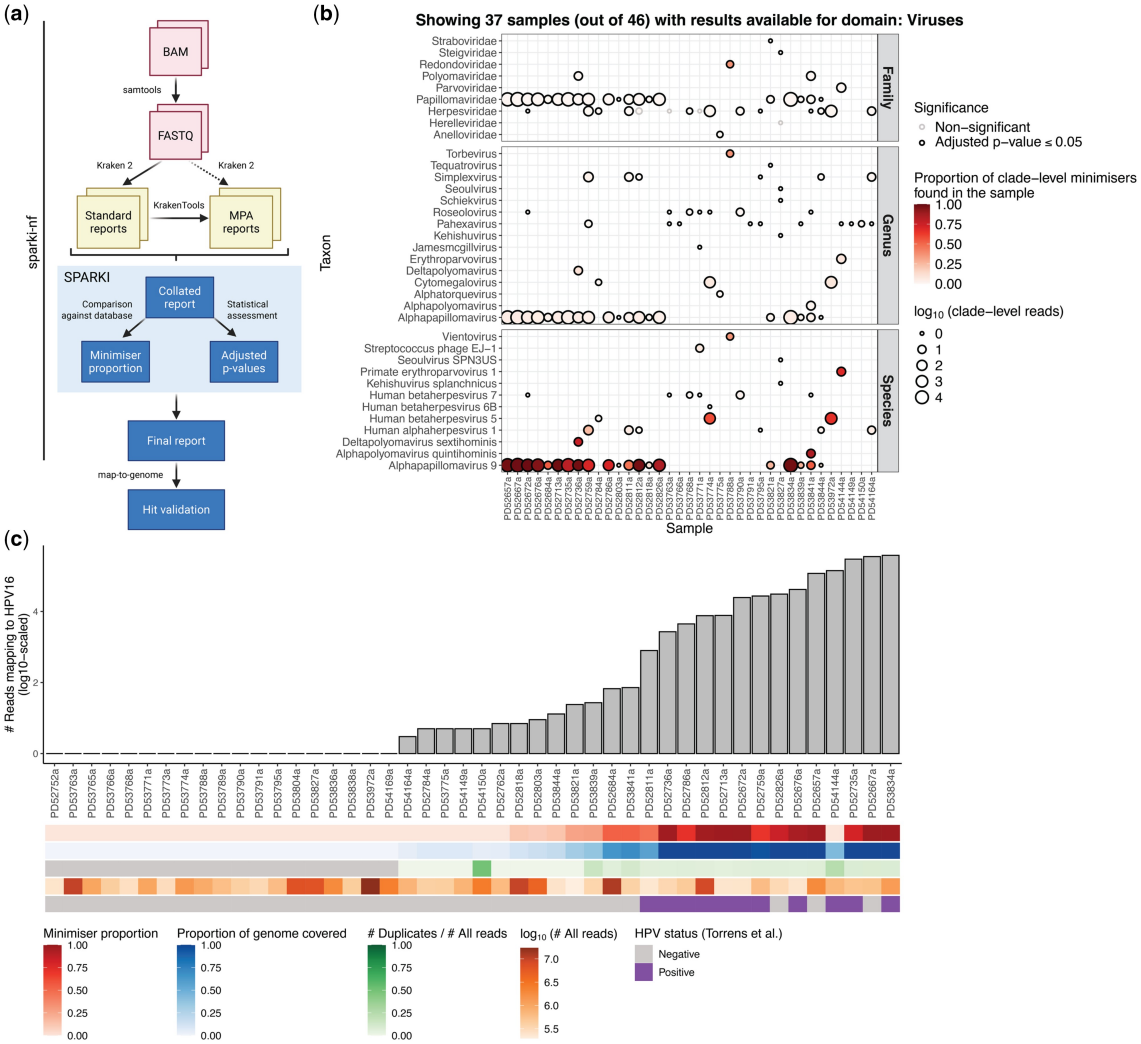
Workflow of an end-to-end pathogen identification analysis with sparki-nf and hit validation with map-to-genome. (A) sparki-nf takes input BAM files and converts them to FASTQ format using samtools (Li *et al.* 2009). In the process, reads mapping to a host genome are removed. Unmapped reads are then run through Kraken 2 (Wood *et al.* 2019) with the ‘--report’ option and ‘--report-minimizer-data’ flag, generating sample-level standard reports. These are used by the KrakenTools’ ‘kreport2mpa.py’ script (Lu *et al.* 2022) to generate sample-level MetaPhlAn2 (MPA)-style reports. Thereafter, SPARKI collates all standard and MPA reports and calculates the minimizer proportion and statistical significance for each taxon. The outputs yielded by SPARKI can then be inspected, and hits of interest can be further explored and validated with map-to-genome: a supplementary pipeline that calculates mapping statistics for alignments between the unmapped reads of a given set of samples against the genomes of specific species provided by the user. This diagram was created with BioRender.com. (B) The whole-genome sequencing data of forty-six oropharyngeal head & neck cancer (HNC) samples was analysed with sparki-nf. The dot plot created with SPARKI shows all samples in which Kraken 2 identified viral reads (columns) and all the viral taxa at the family, genus, and species levels (rows). Dots are coloured by minimizer proportion and dot sizes correspond to the log_10_-scaled number of reads associated with each taxon at the clade level. Additionally, a dot with a black outline denotes that the result for that given taxon in that given sample satisfies adjusted *P*-values ≤ 0.05, as determined by the right-tailed probability of the normal distribution and subsequent multiple comparison correction with the Benjamini & Hochberg method. (C) Validation of SPARKI hits observed in (B) with map-to-genome. Unmapped reads from forty-six HNC samples were aligned against the human papillomavirus 16 (HPV16) reference genome (NCBI RefSeq assembly GCF000863945.3) using BWA-MEM (Li 2013), and mapping statistics were calculated using samtools (Li *et al.* 2009), BEDtools (Quinlan and Hall 2010), and base R functions. The bar plot in (C) shows the log_10_-scaled number of unmapped reads from the HNC samples that aligned to the HPV16 reference genome; the tile plots underneath depict (i) the proportion of Alphapapillomavirus 9 (species of the HPV16 strain) minimizers that were found in the samples, (ii) the proportion of the HPV16 genome that was covered during the mapping process, (iii) the proportion of PCR duplicates across the mapped reads (tiles are grey where no reads aligned to the HPV16 genome), (iv) the total number of reads available for each sample (mapped + unmapped), and (v) the original HPV16 status of each sample provided in the study by Torrens *et al.* (2025) as determined by serology and other methods.

SPARKI (Statistical Process Aimed at Robust Kraken 2 Interpretation, https://github.com/team113sanger/sparki) (Boccacino and Vermes 2025) is an R package and a command line tool developed to help researchers interpret the outputs of Kraken 2. It is available both as a standalone software and as part of an end-to-end pathogen identification pipeline called sparki-nf (https://github.com/team113sanger/sparki-nf) (sparki-nf; Boccacino 2025a) (Fig. 1a), written in NextFlow (Di Tommaso *et al.* 2017). The key sample-level results yielded by SPARKI are, for each taxon, (i) the proportion of minimizers found in the sample (the higher the proportion of minimizers, the more of the organism’s genome is likely present in the sample), and (ii) the statistical significance of the result. The uniqueness of SPARKI is that it leverages minimizer data to assess how plausible it is to find each taxon in a sample given the total number of reads that are being analysed by Kraken 2. The SPARKI framework has been applied to previous studies (Cheema *et al.* 2025, Wong *et al.* 2025), and herein we describe its implementation in detail.

## 2 Implementation

### 2.1 Statistical analysis of pathogen identification results with SPARKI

#### 2.1.1 Input data

SPARKI’s input files are (i) sample-level reports generated by Kraken 2 with the ‘--report’ option and ‘--report-minimizer-data’ flag, and (ii) MetaPhlAn2 (MPA)-style sample-level reports generated by Kraken 2 with the ‘--report’ option and ‘--use-mpa-style’ flag (or by KrakenTools with the script ‘kreport2mpa.py’), herein referred to as standard and MPA reports, respectively.

To use SPARKI as a standalone tool, users must run Kraken 2 and KrakenTools beforehand. Alternatively, users can provide BAM files as input to sparki-nf, our end-to-end pathogen identification pipeline, which will automatically run Kraken 2 and KrakenTools and pipe the reports into SPARKI using the tool’s command line interface.

#### 2.1.2 Report collation

SPARKI can take standard and MPA reports from a cohort of samples and collate them into a single dataframe that retains the original association between samples and their corresponding taxa. Optionally, users can provide a list of samples to omit from the collated dataframe. In the context of a pathogen identification analysis, a host species may be among taxa identified by Kraken 2. The results related to the host species’ own reference genome are therefore removed from the collated dataframe.

MPA reports contain the same information found in standard reports, but they display phylogenetic relationships more clearly. In the report collation process, the results from the MPA reports are reformatted and merged with the standard reports, facilitating the downstream inspection of phylogenetic hierarchies for any taxa alongside the corresponding minimizer data.

#### 2.1.3 Minimizer proportion

Kraken 2 (and consequently, SPARKI) requires a pre-built reference database (DB) to run. Users can generate their own custom DB or download existing DBs that are available online (https://benlangmead.github.io/aws-indexes/k2). A key component of Kraken 2 DBs for running SPARKI is the ‘inspect.txt’ file, which contains a summary of the information included in the DB, such as the total number of minimizers (*M_T_*) associated with each taxon (*T*).

SPARKI leverages *M_T_* to calculate the proportion of minimizers (*p_T_*) for each taxon in a sample. For a given taxon, the proportion is calculated as *p_T_* = (*m_T_/M_T_*), where *m_T_* is the number of unique minimizers associated with that taxon found in the sample. Importantly, the ‘--report-minimizer-data’ flag must be included in the Kraken 2 command that generates standard reports for SPARKI to function, as the minimizer information is a key element for the statistical analysis performed by the software.

#### 2.1.4 Statistical assessment

Assume a sample *S* with a total number of reads (or sample size) *N_S_* is being analysed with Kraken 2. A given number of unique minimizers associated with a taxon *T*, *m_T_*, is identified in *S*, out of the total number of minimizers available for that taxon in the DB, *M_T_*. The probability of identifying the taxon T in *S* (or probability of success) can be given by *P_T_* = (*M_T_/M_DB_*), where *M_DB_* is the total number of minimizers present in the Kraken 2 DB across all taxa. The probability *P* of finding *m_T_* in *S* can be estimated with a binomial distribution, with mean (μ) and standard deviation (σ) given by μ = *N_S_ P_T_* and σ = [*N_S_ P_T_* (1 - *P_T_*)] ^½^, respectively.

Since next-generation sequencing (NGS) experiments usually yield large read numbers, the binomial distribution can then be approximated with a normal distribution. Based on this principle, SPARKI calculates the probability of observing at least *m_T_* in *S* using the right-tailed probability of the normal distribution:


$$P\left(X\geq m_{T}\right)=1-\Phi \;(\left[m_{T}-\mu \right]/\sigma )$$

with Φ representing the cumulative distribution function of the standard normal distribution. This is implemented in R with the pnorm() function from the stats package. The *P*-values obtained are then corrected for multiple comparisons using the Benjamini & Hochberg (BH) method. The correction of *P*-values is performed across taxa of the same taxonomic rank (e.g. *P*-values of species-level taxa are corrected only considering results at the species level). Results satisfying adjusted *P*-value ≤ 0.05 are considered statistically significant.

### 2.2 End-to-end pathogen identification analysis with sparki-nf

To facilitate pathogen identification analysis, we developed a NextFlow pipeline called sparki-nf (https://github.com/team113sanger/sparki-nf) that incorporates samtools (Li *et al.* 2009), Kraken 2 (Wood *et al.* 2019), KrakenTools (Lu *et al.* 2022), and SPARKI, and executes all the necessary steps seamlessly. Starting from BAM files, sparki-nf filters out all reads that were mapped to a host species’ reference genome and subjects the unmapped reads to a Kraken 2 analysis. The sample-level standard reports produced by Kraken 2 are used as input for KrakenTools, which in turn generates sample-level MPA reports. Ultimately, standard and MPA reports are then collated and analysed by SPARKI.

### 2.3 Further validation of SPARKI hits with map-to-genome

To further explore and validate SPARKI hits, we developed a NextFlow pipeline called map-to-genome (https://github.com/team113sanger/map-to-genome) (map-to-genome; Boccacino 2025b). The pipeline takes as input (i) BAM files corresponding to the samples of interest and (ii) FASTA files containing the reference genomes of taxa that SPARKI identified as hits (or of a subset of hits that are of interest to the user); the unmapped reads of each sample are then aligned against each reference genome with BWA-MEM (Li 2013), and PCR duplicate marking and key mapping statistics are calculated for each ‘sample-genome’ pair with samtools (Li *et al.* 2009), BEDtools (Quinlan and Hall 2010), and base R functions. The mapping statistics include: (i) the numbers of mapped and unmapped reads, (ii) the mean and median mapping quality across all mapped reads, (iii) the number of PCR duplicates, and (iv) the proportion of the genome that was covered during the mapping process. The main output generated by map-to-genome is a collated report which contains the mapping statistics of all ‘sample-genome’ pairs.

## 3 Application

To demonstrate SPARKI’s usage, we processed the whole-genome sequencing data of a cohort of 46 oropharyngeal head & neck cancer (HNC) samples (Torrens *et al.* 2025) using sparki-nf (mutographs-hnc-sparki; Boccacino 2025c) (Fig. 1B). HNC in the oropharynx may be associated with human papillomavirus (HPV), and in the original publication this cohort was tested for the presence of HPV16 via serology and other methods. To detect HPV16 here, we set a confidence score of 0.1 for Kraken 2 and performed taxon attribution with a pre-existing 16GB-capped reference DB containing RefSeq sequences from archaea, bacteria, viruses, plasmids, humans, protozoa, fungi, and plants, as well as sequences from the UniVec database, which we downloaded from https://benlangmead.github.io/aws-indexes/k2 (PlusPFP-16, version of March 2023). Figure 1B demonstrates one of SPARKI’s output plots and shows the minimizer proportion for all family-, genus-, and species-level taxa under the ‘viruses’ domain that were identified by Kraken 2 across the HNC samples. Figure 1C demonstrates the outputs produced by map-to-genome, which we used to validate the SPARKI hits related to Alphapapillomavirus 9 (species of the HPV16 strain).

## 4 Conclusion

Here, we introduce SPARKI, an easy-to-use tool that adds a statistical analysis component to the Kraken 2 workflow and facilitates the extraction of meaningful results from the complex outputs it yields. Notably, sparki-nf incorporates SPARKI and enables a seamless end-to-end pathogen identification analysis. We recommend that SPARKI is used primarily as a discovery tool, with its results subsequently validated by orthogonal methods. To support this, we also provide map-to-genome as an option for a deeper validation of results produced by SPARKI and sparki-nf.

## Data Availability

The whole-genome sequencing data from HNC samples (Torrens *et al.* 2025) analysed in this study is available in the European Genome-Phenome Archive (EGA) under the accession number https://ega-archive.org/studies/EGAS00001005450.
